# Ángela Restrepo Moreno 1931-2022

**DOI:** 10.7705/biomedica.7213

**Published:** 2023-08-31

**Authors:** Beatriz L. Gómez

**Affiliations:** Escuela de Medicina y Ciencias de la Salud, Universidad del Rosario, Bogotá, D.C., Colombia Universidad del Rosario Escuela de Medicina y Ciencias de la Salud Universidad del Rosario Bogotá, D.C. Colombia

**Figure:**
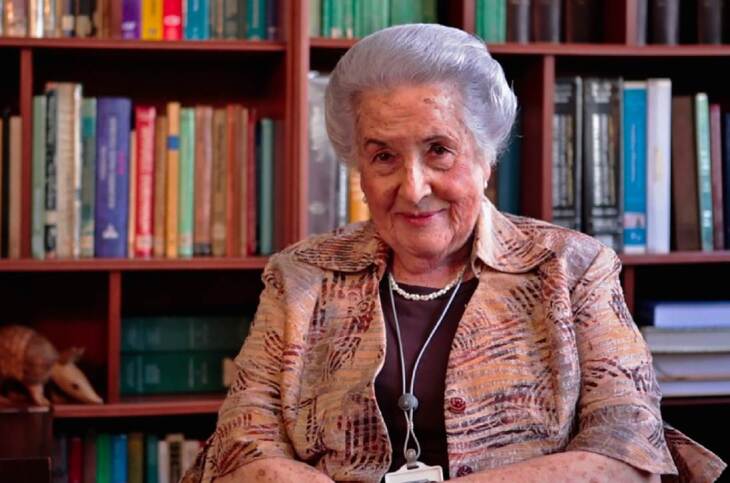

La doctora Ángela Restrepo Moreno nació en Medellín (Colombia) en 1931 y, desde muy joven, dada su curiosidad por descubrir y saber más sobre los pequeños microorganismos causantes de enfermedades -y que se podían observar con el microscopio de su abuelo- escogió la ciencia y la microbiología como el eje de su vida académica. La importancia y la magnitud de la labor científica, pedagógica y humana de “la Doctora”, como la llamábamos con gran respeto, cariño y admiración todos sus alumnos y colegas, la convirtieron en una de las principales figuras de la historia de la ciencia en Colombia. Sus aportes a la micología médica la convirtieron en pionera, en reconocida experta y en “maestra” de muchos en Colombia, en Latinoamérica y en todo el mundo.

La doctora Ángela se graduó en la primera promoción de técnicas de laboratorio clínico del Colegio Mayor de Antioquia de Medellín en 1954. Sus prácticas profesionales las hizo en la Facultad de Medicina de la Universidad de Antioquia, e ingresó al Departamento de Microbiología y Parasitología de esta facultad luego de haberse graduado. Entre 1958 y 1960 cursó una maestría en Ciencias en la Universidad de Tulane (Nueva Orleans, Estados Unidos) y, a su regreso a la Universidad de Antioquia, fundó el Laboratorio de Micología Médica dentro del Departamento de Microbiología y Parasitología, con el cual se inició esta disciplina en el país, y en donde se capacitó un gran número de profesionales.

Su inquietud por aprender más sobre cómo hacer investigación, la llevó de regreso a la Universidad de Tulane para complementar su formación y obtener su doctorado en 1965.

A su regreso a Colombia y a la Facultad de Medicina de la Universidad de Antioquia, se integró de nuevo al Laboratorio de Micología Médica y rodeada de alumnos, colegas y profesores de varias disciplinas, convirtió este laboratorio en un centro de referencia nacional para el diagnóstico, la investigación y la enseñanza de las enfermedades producidas por hongos.

La doctora Restrepo estableció la línea de investigación sobre la paracoccidioidomicosis, causada por el hongo *Paracoccidioides* spp., una micosis a la que dedicó décadas de estudio. Sus investigaciones, en colaboración con sus alumnos y con un grupo importante de profesores del área clínica y de ciencias básicas, incluyeron desde la descripción en 1961 de los primeros casos de esta micosis en Colombia hasta aportes al conocimiento de la paracoccidioidomicosis en todos sus aspectos: su agente etiológico y la búsqueda de su hábitat, la fisiopatología de la enfermedad, el desarrollo y la estandarización de nuevos métodos diagnósticos y de nuevas terapias para su tratamiento.

Otra de las grandes contribuciones de la doctora Ángela en la Universidad de Antioquia fue la creación de la maestría en Microbiología Médica, en asociación con otros profesores del Departamento de Microbiología y Parasitología, donde tuvo la oportunidad de formar la primera generación de micólogos e investigadores profesionales del país. Mediante la coordinación del programa *Latin American Professorship* de la *American Society for Microbiology* -en cuyo convenio vinieron a Colombia destacados microbiólogos estadounidenses a dictar cursos avanzados en diferentes tópicos-, la doctora Restrepo contribuyó a la formación de investigadores en otras áreas de la Microbiología y se logró que muchos estudiantes y profesionales pudieran hacer pasantías y continuar sus estudios en prestigiosas universidades de los Estados Unidos.

Fue profesora titular de la Universidad de Antioquia hasta 1976, y luego de trabajar dos años como subdirectora del Laboratorio Departamental de Antioquia, en 1978 se vinculó a la Corporación para Investigaciones Biológicas (CIB), institución de la que fue cofundadora y de la que fue su directora científica entre 1978 y 2015, es decir, hasta su retiro. La doctora Restrepo, en colaboración con otros distinguidos y reconocidos profesores e investigadores, logró hacer de la CIB uno de los principales centros de investigación biológica no sólo de Colombia, sino de Latinoamérica.

En esta institución, trabajando cooperativamente con un gran número de alumnos de diversas universidades y de profesionales reconocidos y expertos en diversos campos a nivel nacional e internacional, la doctora Ángela continuó su investigacion sobre *Paracoccidioides* spp. y la paracoccidioidomicosis logrando importantes avances y nuevos conocimientos sobre esta enfermedad y su agente etiológico, avances que la llevaron a ser reconocida como la gran experta a nivel nacional e internacional en esta micosis.

Más recientemente, cuando la aplicación de la epidemiología molecular y la genética de poblaciones reveló que el género *Paracoccidioides* se compone, al menos, de cinco especies diferentes, una de las nuevas especies del hongo se denominó *Paracoccidiodes restrepiensis* en su honor.

Aunque la mayor parte de su trabajo se centró en la paracoccidioidomicosis, la doctora Ángela también hizo aportes muy destacados en el diagnóstico y estudio de otras infecciones causadas por hongos, y fue así como alcanzó la plenitud de su producción científica y gran reconocimiento durante los años de trabajo en la CIB.

Su obra científica incluye más de 400 publicaciones nacionales e internacionales y está compuesta por artículos originales, revisiones, ensayos y más de 40 capítulos en libros, muchos de ellos en los más destacados libros de Medicina o de Microbiología a nivel mundial.

La doctora Restrepo recibió dos títulos de doctorado *honoris causa*, uno otorgado por la Universidad Pontificia Bolivariana de Medellín en 1994, y el segundo, por la Universidad de Antioquia de Medellín en 1996. A lo largo de sus más de 50 años de vida científica, recibió muchísimos reconocimientos, premios, distinciones y homenajes (más de 50) por parte de sociedades científicas nacionales e internacionales, universidades e instituciones públicas. Entre estos premios, caben ser destacados tres de ellos otorgados por importantes sociedades científicas internacionales y que ningún científico latinoamericano había recibido antes: el Lucille K. George Award, otorgado por la *International Society for Human and Animal Mycology* (ISHAM) en 1979; el Rodha Benham Award, otorgado por la *Medical Mycology Society of the Americas* en 1990, y la Medical Mycology Medal, otorgada por la *Canadian Society of Mycology* en 1991.

Es de destacar que la doctora Ángela Restrepo fue la única mujer que participó en la “Misión de sabios” creada por el gobierno nacional de Colombia entre 1992 y 1994 para aportar a la construcción e implementación de la política pública de educación, ciencia, tecnología e innovación. Al formar parte de esta comisión, realizó uno de sus grandes sueños, en el que trabajó también por décadas: el descubrimiento y promoción de jóvenes con talento para la investigación.

La doctora Ángela fue miembro activo y honorario de importantes academias científicas colombianas, entre ellas la Academia Colombiana de Ciencias Exactas, Físicas y Naturales, y junto a un número importante de destacados investigadores establecieron una activa colaboración con el Parque Explora de Medellín, logrando incluir en la agenda educativa del parque una serie de actividades de difusión científica, especialmente para niños y jóvenes escolares de escasos recursos. Durante toda su carrera científica, y aun desde su retiro, formó cientos de jóvenes estudiantes y profesionales en el campo de la micología y también apoyó a los de otras disciplinas afines. Sus alumnos hoy se desempeñan en instituciones en Colombia y en otros países, y llevarán siempre la huella imborrable de haber sido sus discípulos. La doctora Ángela solía decir con mucho orgullo que sus alumnos eran sus “mejores condecoraciones” y que eran, además, su familia.

Los últimos años de su vida los consagró a seguir apoyando a la Institución Educativa Ángela Restrepo Moreno, nombrada en su honor. Allí se convirtió en la mentora y guía de todas las actividades científicas, incluso durante los años de la pandemia en la modalidad virtual, animando e inspirando a los jóvenes estudiantes el deseo de continuar sus estudios superiores.

La doctora Ángela era una mujer culta, dotada de una gran sensibilidad por la música, el arte y la literatura. Era una mujer muy generosa y humilde, de buen humor y, sobre todo, era una gran ciudadana con un inmenso amor por su patria. Su inmensa calidez le permitía ser una compañía inigualable en todos los momentos, siempre tenía un mensaje de estímulo, de apoyo, de cariño y de solidaridad para cada persona y en cada momento.

Por su majestuoso legado de ciencia y humanidad, todos sus alumnos, colegas, compañeros de proyectos y todos quienes tuvimos el privilegio y enorme fortuna de conocerla y de ser cercanos a ella, nuestra eterna gratitud, admiración y cariño a la gran científica, a la maestra por excelencia y a la mujer cálida y ejemplar que fue y que seguirá siendo inspiración y modelo para las generaciones presentes y futuras.

